# The *In Vitro* Effect of Acidic-Pepsin on Nuclear Factor KappaB Activation and Its Related Oncogenic Effect on Normal Human Hypopharyngeal Cells

**DOI:** 10.1371/journal.pone.0168269

**Published:** 2016-12-14

**Authors:** Clarence T. Sasaki, Julia Toman, Dimitra Vageli

**Affiliations:** The Yale Larynx laboratory, Department of Surgery, Yale School of Medicine, New Haven, CT, United States of America; National University Singapore Yong Loo Lin School of Medicine, SINGAPORE

## Abstract

**Background:**

Extra-esophageal carcinogenesis has been widely discussed in relation to the chronic effects of laryngopharyngeal reflux and most prominently with pepsin historically central to this discussion. With refluxate known to include gastric (pepsin) and duodenal (bile) fluids, we recently demonstrated the mechanistic role of NF-*κ*B in mediating the preneoplastic effects of acidic-bile. However, the role of pepsin in promoting hypopharyngeal premalignant events remains historically unclear. Here, we investigate the *in vitro* effect of acidic-pepsin on the NF-*κ*B oncogenic pathway to better define its potential role in hypopharyngeal neoplasia.

**Methods:**

Human hypopharyngeal primary cells (HHPC) and keratinocytes (HHK) were repetitively exposed to physiologic pepsin concentrations (0.1 mg/ml) at pH 4.0, 5.0 and 7.0. Cellular localization of phospho-NF-*κ*B and bcl-2 was determined using immunofluorescence and western blotting. NF-*κ*B transcriptional activity was tested by *luc* reporter and qPCR. Analysis of DNA content of pepsin treated HHK and HHPC was performed using Fluorescence-activated-cell sorting assay. To explore a possible dose related effect, pepsin concentration was reduced from 0.1 to 0.05 and 0.01 mg/ml.

**Results:**

At physiologic concentration, acidic-pepsin (0.1 mg/ml at pH 4.0) is lethal to most normal hypopharyngeal cells. However, in surviving cells, no NF-*κ*B transcriptional activity is noted. Acidic-pepsin fails to activate the NF-*κ*B or bcl-2, TNF-α, EGFR, STAT3, and wnt5α but increases the Tp53 mRNAs, in both HHPC and HHK. Weakly acidic-pepsin (pH 5.0) and neutral-pepsin (pH 7.0) induce mild activation of NF-*κ*B with increase in TNF-α mRNAs, without oncogenic transcriptional activity. Lower concentrations of pepsin at varying pH do not produce NF-*κ*B activity or transcriptional activation of the analyzed genes.

**Conclusion:**

Our findings *in vitro* do not support the role of acidic-pepsin in NF-*κ*B related hypopharyngeal carcinogenesis.

## Introduction

The American Cancer Society estimates approximately 3,000 cancers will start in the hypopharynx in 2016 but only 53% of cases even at early stages will survive 5-years [1,2]. Because of its anatomic site several environmental factors are considered to be risk factors, including tobacco and alcohol or their combinations [3,4]. Esophageal and specifically llaryngopharyngeal reflux disease (ERD or LPR), are also considered potential risk factors in hypopharyngeal carcinogenesis [5–7]. Gastroesophageal reflux is suggested by others to be an independent risk factor for laryngopharyngeal cancer [8]. Furthermore, the clinical prevalence and magnitude of LPR and its association with non-oesophageal cancer may be greater than it is already considered [9]. We recently provided evidence *in vitro* and *in vivo* of the potential role of gastroduodenal fluid and specifically of bile at acidic pH (≤4.0) in hypopharyngeal neoplasia, mediated by the NF-*κ*B activated pathway [10,11].

According to prior studies of 24-hour ambulatory pH monitoring in the pharynx of patients, a drop below pH 4.0 is not uncommon and is considered diagnostic of a reflux event [12]. Moreover, some investigators consider detection of a pH in the pharynx less than 4.0 more than 1% of the study time to be pathological [13], whereas in a large population of normal subjects a pH less than 4.0 may be occasionally observed [14]. Pepsin, a protease secreted by gastric chief cells, has clearly been shown to be a component of the refluxate that is capable of extending supraesophageally to the larynx [15]. Pepsin is active at acidic pH (≤4.0), while in less acidic environment the catalytic activity of pepsin gradually decreases with increasing pH until it is fully denatured and irreversibly inactivated at pH 8.0 [16].

The mechanistic role of NF-*κ*B between chronic inflammation and carcinogenesis is well demonstrated [17], and its role may be crucial in head and neck carcinogenesis. In head and neck squamous cell carcinoma (HNSCC) NF-*κ*B is often upregulated from premalignant lesions to invasive cancer [18–20]. Specifically, it has been shown that dysregulated signaling networks common in HNSCC include aberrant *NF-κ*B activation, contributing to the expression of genes that can modulate apoptotic resistance and cell survival like bcl-2 [21], EGFR [22,23], STAT3 [24,25] and wnt5α [26,27]. Additionally, elevated function of NF-*κ*B has been associated with the activation of TNF-α, while promoting cREL nuclear translocation [28] that again correlates with tumor progression [29,30].

The purpose of this study is to clarify whether constitutive stimulation of human hypopharyngeal primary cells (HHPC) and immortalized human hypopharyngeal keratinocytes (HHK) with acidic (pH 4.0), weakly acidic (pH 5.0) or neutral (pH 7.0) pepsin is capable of inducing transcriptional activation of anti-apoptotic genes, including bcl-2, cell signaling TNF-α, oncogenic EGFR, STAT3, wnt5α, Tp63 or altering the expression of cell cycle control-related Tp53 linked to HNSCC [18–32]. If acidic-pepsin contributes to the activation of the NF-*κ*B oncogenic pathway, its effects with acidic-bile could exert an additive or synergistic effect on neoplastic events.

## Materials and Methods

### Normal human hypopharyngeal cell cultures

Human hypopharyngeal primary cells (HHPC) were obtained from Celprogen Inc. CA, USA. The HHPC were plated in non-coated flasks and were grown in Human Hypopharyngeal Normal Cell Culture Media with Serum (Celprogen Inc. CA, USA), at 37°C in humidified air and 5% CO_2_. Cells were sub-cultured and media were gradually replaced by Serum Free Media (Celprogen Inc. CA, USA).

We also established a telomerase-immortalized human hypopharyngeal cell line (HHK), by expression of *h*TERT, extending its life span without altering the characteristic phenotypic properties of the cells, as previously described [10,33]. HHK, were grown in keratinocyte serum free basal medium (KGM-2 SF, Gibco^®^) supplemented by L-Glutamine, BPE, *h*EGF and gentamicin (Gibco^®^), at 37°C in humidified air and 5% CO_2_.

### Treatment conditions

We performed a repetitive exposure of HHPC (2nd passage) and HHK (4th passage) to physiologic concentration of pepsin fluid, in accordance with concentrations clinically known in reflux disease [34]. Cells were exposed for 10–15 min, 3 times per day for 4–5 days, in line with a previously established *in vitro* model demonstrating that gastroduodenal fluid can induce NF-*κ*B activation and transcriptional activation of a related oncogenic pathway in human hypopharyngeal normal cells, during a period of 4–5 days of repetitive exposure [10]. Our experimental exposure included application of 0.01, 0.05 and 0.1 mg/ml of porcine pepsin (Sigma Aldrich^®^), as previously described [35], in DMEM/F12 10% FBS, and using a pH meter brought to a (a) pH of 4.0 (acidic-pepsin) with 1M HCl, the cut-off of LPR episodes [14,36,37], (b) pH of 5.0 (weakly acidic-pepsin) with 1M HCl, (c) pH of 7.0 (neutral-pepsin), and (d) pH 8.0 with 10 M NaOH, incubated at 37°C for 30 min and then reduced to pH 7.0 (using 1 M HCl), as previously described [38], at which pepsin is irreversibly inactive. Porcine pepsin (A) is commercially available and has similar activity to human pepsin (C) [39].

We also used control media for the corresponding experimental cultures consisting of the same fluid without pepsin at pH 4.0 (acid-control), 5.0 (weakly acidic-control), and 7.0 (neutral-control), while an untreated culture was used as a reference control.

The experimental and control media were removed and replaced with KGM-2 SF at pH 7.2 until the next exposure cycle. At the end of treatment procedures media were removed and cells or cell extracts were analyzed.

### Immunofluorescence assay

The primary HHPC and the immortalized HHK were grown on multiwall chambers and underwent repeated exposure to experimental and control fluids. We performed an immunofluorescence assay, as we previously described [10], using 1:100 NF-*κ*B primary (anti-phospho-p65 S529; 44-711G; Invitrogen^TM^, Thermo Fisher Scientific), and secondary Alexa Flour^®^ 488 IgG (Abcam). Cells were mounted using Prolong^®^ Gold mountant with DAPI (Life Technologies) for nuclear staining. Slides were examined using a Zeiss Confocal microscope and images were captured using the Zen imaging software (Carl Zeiss; Germany).

### Western blotting

Total cytoplasmic and nuclear protein expression levels of the cultured HHPC and HHK were determined by western blot analysis, as we previously described [10]. We used NF-*κ*B (p65) (F-6; Santa Cruz), phospho-NF-*κ*B (p65 S529) (44-711G; Invitrogen^TM^, Thermo Fisher Scientific), phospho-IKB-α Ser32/36 (5A5; Cell Signaling), and bcl-2 (C-2; Santa Cruz). We also used β-actin (C4; Santa-Cruz), for cytoplasmic and nuclear extract normalization. Protein levels were quantified by Gel-imaging system (*BIO-RAD*), in each nuclear and cytoplasmic cellular compartment, and expression levels were estimated by Image Lab 4.1 analysis software 4, *BIO-RAD*). PARP control was omitted as we focused on NF-*κ*B induced oncogenicity rather than apoptosis.

### Luciferase assay

We performed a luciferase assay to monitor the activity of the NF-*κ*B in the pepsin treated cultured HHPC and HHK using Dual-Glo® Luciferase Assay system (Promega Corporation), Lipofectamine^®^ 2000 (Invitrogen^TM^), and NF-*κ*B responsive element (3kB conA-luc) and control (conA-luc), as we previously described [10]. NF-*κ*B activity values were expressed as ratios of mean values calculated in experimentally treated HHPC and HHK that were determined by comparing values (NF-*κ*B reporter-3kB conA-luc /reference control-conA-luc), against the mean value for their corresponding controls.

### Quantitative real time PCR

We performed real-time qPCR analysis (Bio-Rad; thermal cycler CFX96^TM^) to evaluate mRNA levels of the target genes, RELA (p65), c-REL, bcl-2, TNF-α, Tp63, EGFR, STAT3, wnt5α and Tp53, and the reference housekeeping gene, *h*GAPDH, using specific primers for human genome (QuantiTect^®^ primers assay, Qiagen) (Table 1), and iQ^TM^ SYBR^®^ Green Supermix (*BIO-RAD*), as we previously described [10]. Assays were performed in 96 well-plates, in triplicate for each sample, and data were analyzed by CFX96 Manager^TM^ software. Relative mRNA expression levels were estimated for each target gene relative to reference gene (ΔΔ*C*_*T*_).

**Table 1:** Human genes analyzed by real-time qPCR, in normal human hypopharyngeal cells.

Gene	Detected transcripts	Amplicon length (bp)
***h*GAPDH**	NM_001256799, NM_002046	95
**bcl-2**	NM_000633	116
**EGFR**	NM_005228,	80
	NM_201282–4	
**REL**	NM_002908	117
**RELA**	NM_001145138, NM_001243984–5, NM_021975	107
**wnt5A**	NM_001256105, NM_003392	105
**Tp63**	NM_001114980, NM_003722	130
**TNF**	NM_000594	98
**STAT3**	NM_003150,	95
	NM_139276	
**Tp53**	NM_000546, NM_001126112–8, NM_001276695–99, NM_001276760–1,	112

### Statistical analysis

Statistical analysis was performed using GraphPad Prism 6 software. Comparison between protein or mRNA expression of different experimental and control groups was performed using ONE-WAY ANOVA (Friedman or Kruskal-Wallis and Dunn’s multiple analysis test; *p*-values<0.01) while the correlation coefficient between expression levels of different groups was estimated using *Pearson* correlation (*p*-values<0.05). Specifically, we used *Pearson* analysis to identify a positive or inverse correlation among the relative mRNA expressions (pepsin/control) of the analyzed genes, in the three different groups (acidic, weakly acidic and neutral pH). Calculation and real-time PCR analysis data were performed by Bio-Rad Thermal cycler CFX96 Manager^TM^ software (*BIO-RAD*).

### Fluorescence-activated cell sorting assay (FACS)

A fluorescence-activated cell sorting assay (FACS) was used in order to analyze the DNA content of the pepsin-treated HHPC and HHK. The HHK and HHPC that underwent repetitive exposure to pepsin and control fluids (under similar conditions used in the experimental protocol) were harvested and fixed in ice-cold 70% ethanol over night at -20°C. Ethanol was removed by centrifugation and the cells were rehydrated in PBS and pelleted. The pellets were resuspended in 25 μg/ml propidium iodide (Sigma) in PBS containing 100 μg/ml RNase A (Invitrogen) and stained for 30 min at room temperature. The DNA content was analyzed by FACSCalibur^TM^ flow cytometer (BD Biosciences). Samples were gated on the single cell population, and 10,000 cells were collected for each sample.

## Results

### Acidic-pepsin does not induce NF-*κ*B activation or bcl-2 overexpression in treated HHK and HHPC

**Fig 1:**
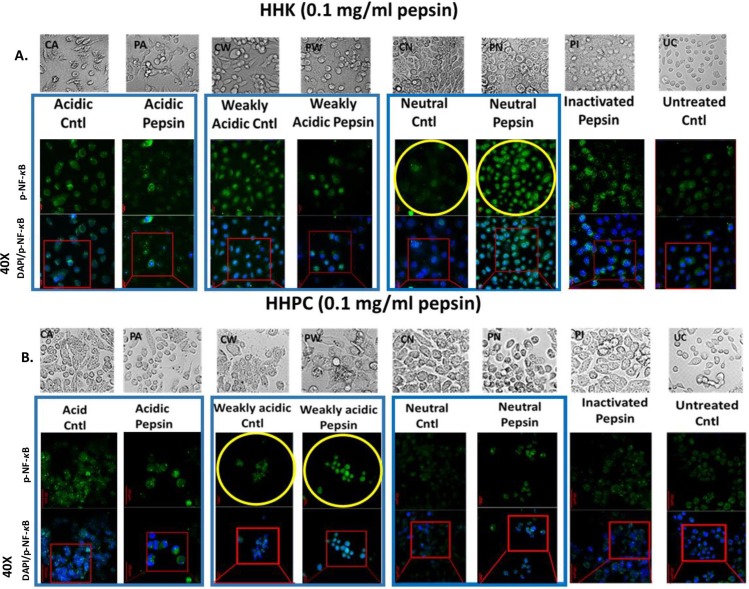
Effect of pepsin in p-NF-κB nuclear localization, in human hypopharyngeal keratinocytes (HHK) and human hypopharyngeal primary cells (HHPC). Immunofluorescence (IF) staining of phospho-NF-κB (p-p65 S529) is demonstrated in **(A)** HHK and **(B)** HHPC treated by 0.1 mg/ml pepsin at different pH (acid, pH 4.0; weakly acidic, pH 5.0 and neutral, pH 7.0) and corresponding controls. Acidic-pepsin treated HHK and HHPC demonstrates mainly cytoplasmic localization of p-NF-κB. In contrast, neutral-pepsin treated HHK and weakly acidic-pepsin treated HHPC results in an intense nuclear localization of p-NF-κB.

To investigate whether pepsin affected NF-*κ*B (p65) localization in normal human keratinocytes we performed an immunofluorescence assay (IF). Repetitive exposure of both HHK and HHPC to acidic-pepsin (active pepsin) did not induce nuclear localization of p-NF-*κ*B. However, neutral-pepsin led to NF-*κ*B activation in treated HHK, while weakly acidic-pepsin induced minimal increase of NF-*κ*B activated levels in treated HHPC, compared to controls (Fig 1).

**Fig 2:**
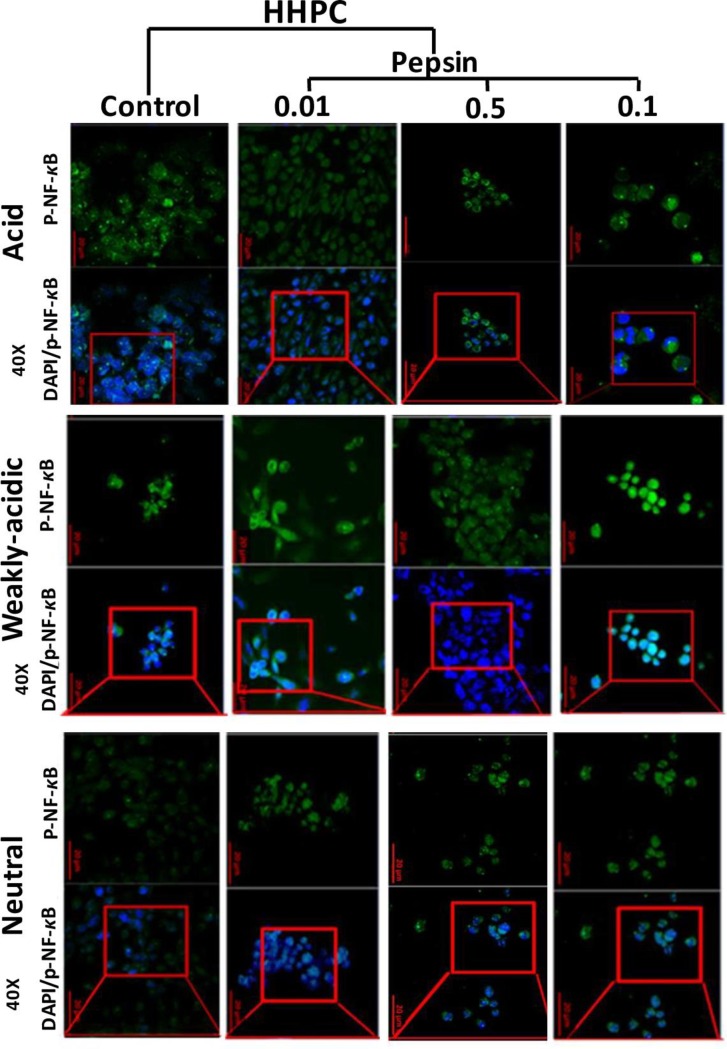
Varying pepsin concentrations at different pH do not affect the nuclear localization of p-NF-κB, in human hypopharyngeal primary cells (HHPC). Immunofluorescence (IF) staining of phospho-NF-κB (p-p65 S529) is demonstrated in HHPC treated by 0.01, 0.5 and 01 mg/ml of pepsin at different pH (acid, pH 4.0; weakly acidic, pH 5.0 and neutral, pH 7.0) and corresponding controls.

Specifically, both HHK and HHPC repetitively exposed to 0.1 mg/ml of acidic-pepsin (pH 4.0) demonstrated cytoplasmic p-NF-*κ*B (p-p65 S529) staining with a few sporadic cells that were positive for p-p65 nuclear staining (Fig 1A and 1B). In contrast, HHK exposed to neutral-pepsin (pH 7.0) (Fig 1A) and HHPC exposed to weakly acidic fluids (pH 5.0) and particularly in combination with pepsin (Fig 1B) exhibited a more intense nuclear p-p65 IF-staining. Neutral or acidic-control produced sporadic cells that were positive for both cytoplasmic and nuclear p-p65 IF-staining, while both HHK and HHPC treated by inactivated pepsin, as well as untreated controls, demonstrated cytoplasmic p-p65 staining only. We further demonstrated that lower concentrations of pepsin (0.05 and 0.01 mg/ml) did not induce nuclear localization of NF-*κ*B in treated HHPC (Fig 2).

**Fig 3:**
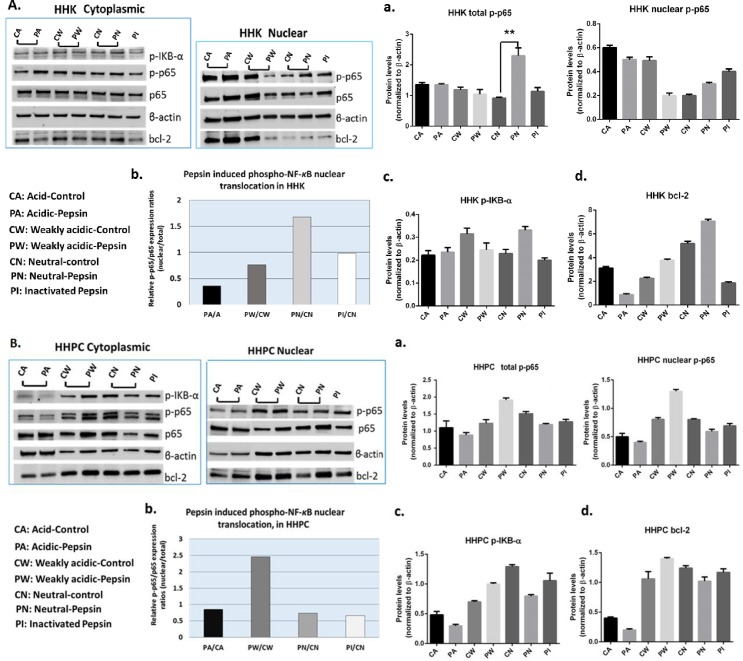
Effect of pepsin in NF-κB activation and bcl-2 overexpression, in human hypopharyngeal keratinocytes (HHK) and human hypopharyngeal primary cells (HHPC). Western blot analysis for NF-κB (p65), p-NF-κB (p-p65 S529), p-IKB-α (Ser32/36) and bcl-2 protein levels demonstrates that acidic-pepsin does not induce NF-κB activation or bcl-2 overexpression in treated **(A)** HHK and **(B)** HHPC. Columns of the graph correspond to **(a)** total and nuclear p-NF-κB (p-p65) levels, **(b)** pepsin-induced nuclear p-NF-κB (p65 S529) translocation values (p-p65/p65 nuclear/total ratios), **(c)** cytoplasmic p-IKB-α and **(d)** cytoplasmic/nuclear bcl-2 protein levels (ONE-WAY ANOVA; Kruskal-Wallis; GraphPad Prism 6.0). Mean ±SD of three independent experiments.

To further analyze NF-*κ*B activation and bcl-2 expression levels under pepsin treatment, we performed western blot analysis in nuclear and cytoplasmic protein fractions of HHK and HHPC exposed to pepsin fluids and corresponding controls (Fig 3). Our data indicate that *in vitro* exposure of HHK and HHPC to active pepsin (pH 4.0) did not induce NF-*κ*B activation or bcl-2 accumulation in the cytoplasm. However, pepsin at higher pH (5.0 and 7.0) resulted in higher nuclear NF-*κ*B levels, compared to their corresponding controls.

Specifically, we showed that HHK or HHPC exposed to acidic-pepsin produced lower total and nuclear p-p65 levels, compared to acid-control (Fig 3A-a and 3B-a), an observation confirmed by the calculated nuclear/total p-p65/p65 ratios (p-NF-*κ*B nuclear translocation) (Fig 3A-b and 3B-b). In contrast, HHK treated by neutral-pepsin and HHPC exposed to weakly acidic-pepsin exhibited higher nuclear and total p-p65 levels, compared to their corresponding controls (Fig 3A-a and 3B-a), an observation also confirmed by the calculated nuclear/total p-p65/p65 ratios (p-NF-*κ*B nuclear translocation) (Fig 3A-b and 3B-b). We also observed that HHK exposed to neutral-pepsin and HHPC exposed to weakly acidic-pepsin exhibited the highest cytoplasmic p-IKB-α ratios (pepsin/control), supporting elevated NF-*κ*B activated levels in treated cells (Fig 3A-c and 3B-c) [40]. We additionally showed that both HHK and HHPC treated by acidic-pepsin produced lower cytoplasmic/nuclear bcl-2 ratios, compared to acid-control, while both neutral or weakly acidic-pepsin did not induce significant cytoplasmic bcl-2 accumulation in treated HHK or HHPC, compared to their corresponding controls (Fig 3A-d and 3B-d).

*Pearson* correlation revealed that NF-*κ*B activated levels were related to p-IKB-α levels (*r* = 0.96458, *p* = 0.0355) in HHPC but not to bcl-2 ratios. Additionally, *Pearson* analysis did not reveal a significant correlation between NF-*κ*B activation and p-IKB-α levels or bcl-2 overexpression, in treated HHK.

### Acidic-pepsin does not induce NF-*κ*B transcriptional activity

**Fig 4:**
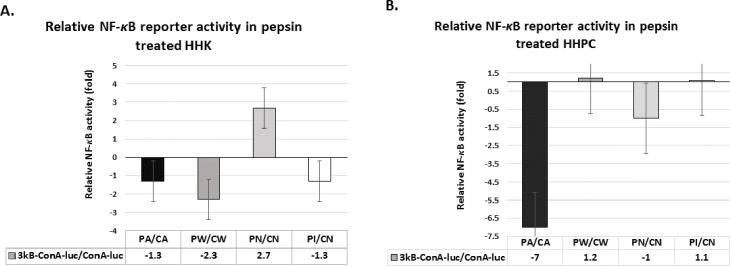
**Luciferase assay demonstrates the effect of pepsin in NF-κB transcriptional activity in (A) HHK and (B) HHPC.** Acidic-pepsin (pH 4.0) does not induce NF-κB transcriptional activity in treated human normal hypopharyngeal cells. Columns represent NF-κB relative transcriptional activity (NF-κB responsive luciferase reporter/control luciferase reporter). 3kB-ConA-luc: NF-κB responsive luciferase reporter; ConA-luc: control luciferase reporter; 3kB-ConA-luc/ConA-luc: NF-κB responsive luciferase reporter/control luciferase reporter. CA: Acid-Control; PA: Acidic-Pepsin; CW: Weakly Acidic-Control; PW: Weakly Acidic-Pepsin; CN: Neutral-Control; PN: Neutral-Pepsin; PI: Inactivated-Pepsin.

To investigate pepsin induced NF-*κ*B transcriptional activity we used NF-*κ*B luciferase reporter (Fig 4). The luciferase assay did not demonstrate increased NF-*κ*B activity in acidic-pepsin treated HHK (Fig 4A) or HHPC (Fig 4B) relative to acid or neutral controls. However, we observed minimal increase of NF-*κ*B luciferase reporter activity in HHPC treated by weakly acidic-pepsin and in HHK exposed to neutral-pepsin, relative to their corresponding controls. We also observed that HHK exposed to weakly acidic-pepsin and HHPC treated by neutral-pepsin exhibited lower levels of NF-*κ*B luciferase reporter activity, relative to their controls, while inactivated-pepsin did not induce NF-*κ*B transcriptional activity.

**Fig 5:**
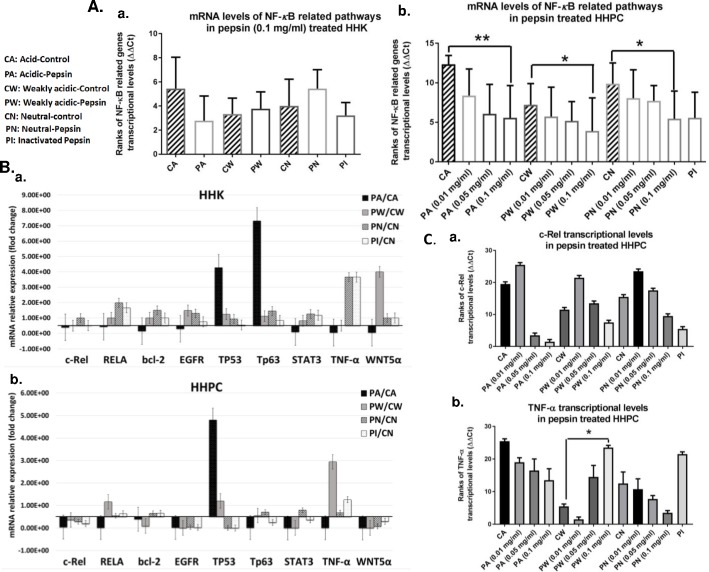
Quantitative real-time PCR demonstrates the effect of pepsin in transcriptional activation of NF-κB and related oncogenic pathway, in treated human hypopharyngeal keratinocytes (HHK) and human hypopharyngeal primary cells (HHPC). **A**. Transcriptional levels of all the analyzed NF-κB related genes (normalized to hGAPDH) are demonstrated in pepsin and control treated **(a)** HHK and **(b)** HHPC. Transcriptional levels of all the analyzed genes were significantly lower in HHPC exposed to 0.1 mg/ml of pepsin, compared to their corresponding controls. (ONE-WAY ANOVA, by Freidman; Dunn’s multiple comparisons test; *p<0.01; **p<0.001). **B**. Columns correspond to relative (pepsin/control) normalized transcriptional levels of each bcl-2, EGFR, c-REL, RELA(p65), Tp63, STAT3, wnt5α, TNF-α, and Tp53 gene, in treated **(a)** HHK and **(b)** HHPC. **C**. Columns correspond to transcriptional levels of **(a)** c-REL and **(b)** TNF-α in HHPC exposed to varying pepsin-concentrations and pH, and corresponding controls. (ONE-WAY ANOVA, Kruskal-Wallis; Dunn’s multiple comparison test; GraphPad Prism 6 software). (CA: Acid-Control; PA: Acidic-Pepsin; CW: Weakly Acidic-Control; PW: Weakly Acidic-Pepsin; CN: Neutral-Control; PN: Neutral-Pepsin; PI: Inactivated-Pepsin).

To evaluate pepsin-induced NF-*κ*B related gene expression profiles we performed real-time qPCR analysis in whole transcriptomes of pepsin-treated HHK and HHPC and their corresponding controls. Repetitive exposure of both HHK and HHPC to pepsin and especially to active pepsin (pH 4.0) did not induce significant transcriptional activation of NF-*κ*B transcriptional factors (TFs), RELA(p65) and c-REL, and NF-*κ*B related genes, including anti-apoptotic bcl-2, or the oncogenic TNF-α, EGFR, STAT3, wnt5α, Tp63 and Tp53 (Fig 5). Specifically, we showed that 0.1 mg/ml of acidic-pepsin did not induce transcriptional activation of the NF-*κ*B related genes in treated HHK (Fig 5A-a) and HHPC (Fig 5A-b), compared to controls. In contrast, acidic-pepsin resulted in significantly lower levels of all the analyzed genes in treated HHPC, compared to their corresponding controls (acid-control vs. acidic-pepsin, *p* = 0.0012; weakly acid-control vs. weakly acidic-pepsin, *p* = 0.0107; neutral-control vs. neutral-pepsin, *p* = 0.0251, by Friedman) (Fig 5A-b). However, we showed that acidic-pepsin treated HHK and HHPC, produced increase in Tp53 mRNA levels, compared to acid alone (4 and 5-fold, respectively) (Fig 5B-a and 5B-b). We also observed that HHK exposed to acidic-pepsin exhibited 7-fold increase of Tp63 mRNAs, compared to acid alone (Fig 5B-a).

Moreover, we showed that HHK or HHPC exposed to neutral or weakly acidic-pepsin exhibited a relative increase of TNF-α mRNAs, compared to their corresponding controls (3.5 and 3-fold, respectively) (Fig 4B-a and 4B-b), supporting the observation of a mild NF-*κ*B activation in the corresponding treated cells, using IF or western blot assays. We also showed that HHK exposed to weakly acidic-pepsin exhibited a relative increase of wnt5α mRNAs, compared to its corresponding control (4-fold) (Fig 5B-a). Finally, we observed that irreversibly inactivated-pepsin had no effect on the NF-*κ*B related genes (Fig 5B-a and 5B-b).

In order to explore the concentration dependent effect of pepsin, we performed real-time qPCR analysis on HHPC treated by 0.05 and 0.01 mg/ml of acidic, weakly acidic, neutral and inactivated-pepsin. We observed that 0.05 and 0.01 mg/ml of pepsin had no effect on transcriptional activation of the NF-*κ*B related genes (Fig 5A-b). Interestingly, exposure to 0.1 and 0.05 mg/ml of acidic-pepsin led to reduced c-REL mRNAs, compared to acid alone (Fig 5C-a). In contrast, low concentration (0.01 mg/ml) of acidic or weakly acidic-pepsin induced mild increase of c-REL, in treated HHPC. Exposure of HHPC to 0.05 mg/ml of weakly acidic-pepsin resulted in mild increase of TNF-α. In contrast, acidic or neutral-pepsin led to reduced expression, compared to their corresponding controls (Fig 5C-b).

### Correlations between NF-*κ*B and related genes expression in pepsin-treated HHK and HHPC

**Fig 6:**
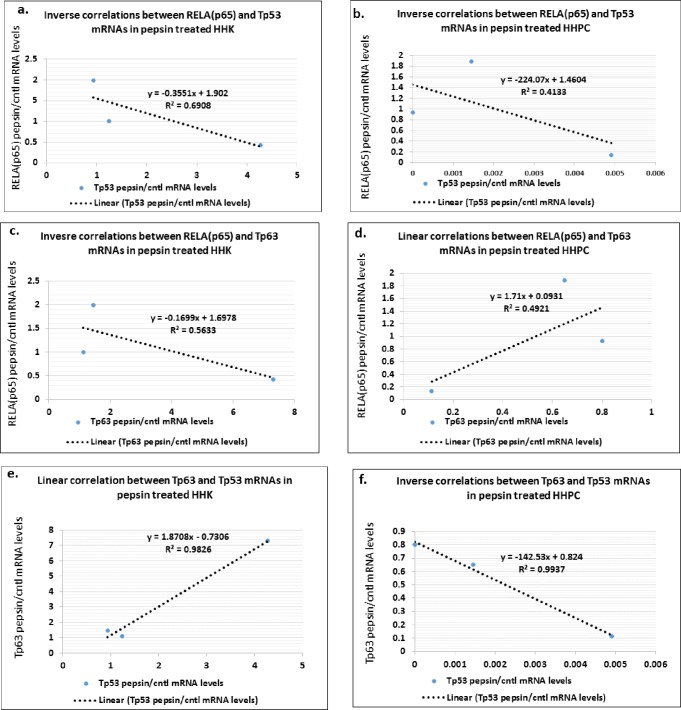
Diagrams show significant correlations by Pearson of relative (pepsin/cntl) expression between NF-κB and related genes in treated HHK and HHPC. Correlation is demonstrated between **(a)** RELA(p65) and Tp53 mRNAs in HHK, **(b)** RELA(p65) and Tp53 mRNAs in HHPC, **(c)** RELA(p65) and Tp63 mRNAs HHK, **(d)** RELA(p65) and Tp63 mRNAs in HHPC, as well as between **(e)** Tp53 and Tp63 mRNAs in HHK and **(f)** Tp53 and Tp63 mRNAs in HHPC (by Pearson; p-value<0.05).

We performed *Pearson* analysis in order to identify any positive or inverse correlations between NF-*κ*B and related genes expression in pepsin-treated HHK and HHPC (Fig 6). We noted an inverse but not statistically significant correlation between RELA(p65) and Tp53 relative expression in both HHK and HHPC (*r* = -0.64288, *r* = -0.83112, respectively), by *Pearson* (Fig 6A and 6B). We additionally observed an inverse correlation between RELA(p65) and Tp63 relative expression in HHK (*r* = -0.75054), but a positive linear correlation in HHPC (*r* = 0.7015) (Fig 6C and 6D). Finally, pepsin-induced Tp53 and Tp63 relative expression showed a significant linear correlation in HHK (*r* = 0.99127, *p* = 0.01), but a significant inverse correlation in HHPC (*r* = -0.997, *p* = 0.003) (Fig 6E and 6F).

### Combination of pepsin and acid is toxic for HHK and HHPC

**Fig 7:**
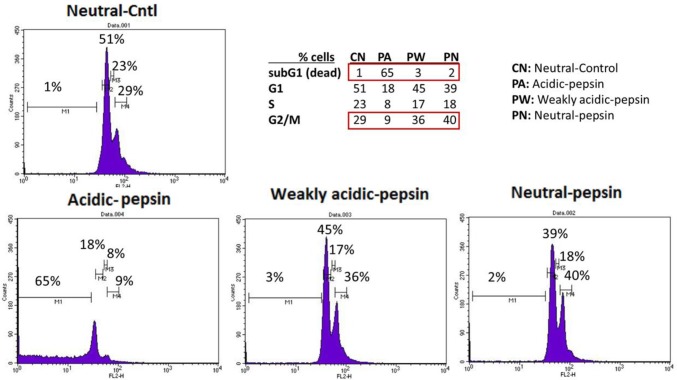
Fluorescence-activated cell sorting assay (FACS) demonstrates pronounced cell death (subG1 population) in acidic-pepsin treated human hypopharyngeal keratinocytes (HHK). FACS shows that less acidic pH (≥5.0) is less toxic to pepsin-treated HHK, resulting in higher percentage (%) of cells in G2/M phase relative to acidic-pepsin (pH 4.0).

We found that acidic-pepsin (0.1 mg/ml at pH 4.0) significantly reduced the survival of HHK, while cells exposed to weakly acidic-pepsin, neutral-pepsin or inactivated-pepsin showed stable viability comparable to neutral-control (Fig 7). Specifically, fluorescence-activated cell sorting assay (FACS) in HHK demonstrated that acidic-pepsin induced pronounced cell death (65% of subG1 population). However, weakly acidic (pH 5.0) or neutral (pH 7.0) pepsin had little effect on cell viability with higher percentage of cells in G2/M phase, relative to acidic-pepsin (pH 4.0).

## Discussion

Pepsin is considered an important clinical marker for the presence of gastroesophageal refluxate when found in the upper aerodigestive tract [7,34]. As a result, many have attempted to link pepsin to carcinogenesis of the laryngopharynx [7,35,38,41]. However, the effect of pepsin to induce early events linked to extra-esophageal neoplasia has never been adequately clarified and its support has been historically divergent despite a growing body of evidence identifying gastroesophageal and laryngopharyngeal reflux as independent risk factors in the development of laryngopharyngeal cancer [8]. As evidence, the role of combined pepsin and acid as a risk factor of neoplasia is supported by Adams and Heintz [41]. On the other hand, Del Negro concludes that pepsin is not carcinogenic in rats [42], while other investigators suggest that non-acidic-pepsin can induce cell proliferation in laryngopharyngeal squamous carcinoma FaDu cell lines [35,38]. Whereas previous *in vitro* studies described the results of a single exposure of hypopharyngeal cancer cell lines to pepsin fluid, for a prolonged period of time (1 to 24 hours) at pH higher than or equal to 7.0, we selected normal human hypopharyngeal primary cells and normal hypopharyngeal keratinocytes treated by test fluids for shorter duration (10–15 min) and over repeated exposures to mimic more closely clinical LPR events. [37].

With regard to the range of acidified pepsin selected, test solutions below pH 4.0 were not selected because of pronounced toxicity and cell death (Fig 7). Similarly, test solutions above pH 7.0 were omitted because pepsin above pH 7.0 is enzymatically inactive [16].

Specifically, our novel findings demonstrate that a combination of pepsin and acid (pH 4.0) in normal human hypopharyngeal cells is not capable of inducing NF-*κ*B activation, nor can it induce significant bcl-2 overexpression or promote transcriptional activation of genes related to oncogenic function. Specifically, the chronic exposure of HHPC to a physiologic concentration of pepsin (0.1 mg/ml) at acidic pH (4.0) produces lower NF-*κ*B transcriptional activity compared to acid or neutral-control. Real-time qPCR reveals that acidic-pepsin expresses lower mRNA levels of the analyzed NF-*κ*B transcriptional factors, RELA(p65) and c-REL [18–20,28,31], anti-apoptotic bcl-2 [21,32], cell signaling TNF-α [28–30,43], oncogenic STAT3, an important molecule involved in NF-*κ*B related tumorigenic phenotypes [20,23–25,44], EGFR, a common genetic event in HNSCC [20,22,23], and wnt5α, upregulated in epithelial to mesenchymal transition [27].

The repetitive exposure of HHPC and HHK to acidic-pepsin maintains elevated transcriptional levels of Tp53 compared to acid alone, whereas Tp53 is considered a fundamental mediator of diverse cellular stress factors sensitizing cells to death, either inducing apoptosis directly or enhancing cell death in ligand-rich environments [45]. Furthermore, we show an inverse correlation between pepsin induced transcriptional levels of NF-*κ*B transcriptional factor, RELA(p65) and Tp53, in both HHK and HHPC (Fig 5A and 5B), in line with previously identified NF-*κ*B and Tp53 inverted phenotypes in inflammatory conditions and related cancers [46].

Pepsin exposures at less acidic environment (pH 5.0 and 7.0) result in a mild activation of NF-*κ*B, associated with a relative increase of TNF-α mRNAs, although unassociated with oncogenic function, providing further evidence that neutral-pepsin may not be a direct risk factor for laryngopharyngeal tumorigenesis. The mild increase of NF-*κ*B activity may be related to a non-specific stress reaction [47], at pH between 4.0 to 7.0.

Other minor differences in response of HHPC and HHK to pepsin deserve discussion. Our results from western blotting and immunofluorescence assay differentially describe nuclear p-p65/p65 (S529) translocation patterns between normal primary epithelial cells (HHPC) and immortalized keratinocytes (HHK) treated with pepsin at less acidic pH (pH 5.0 and 7.0). Primary epithelial cells are more capable of responding to external stimuli such as pepsin at pH 5.0 than immortalized keratinocytes, producing mild NF-*κ*B activation, again without evidence of oncogenic function (Figs 3B, 4B and 5Bb) [48]. On the other hand, immortalized keratinocytes HHK respond to neutral-pepsin (pH 7.0) by minimal activation of NF-*κ*B (Figs 3A, 4A and 5Ba). We also note that HHK responds to weakly acidic-pepsin by an increase in wnt5α, but without co-activation of NF-*κ*B, TNF-α, STAT3, EGFR or Tp53 and Tp63, supporting a wnt5α function in this setting as an important key molecule toward epithelial differentiation rather than oncogenesis [49].

Truong et al have shown that p63 is required for both cell proliferation and differentiation of keratinocytes [50]. Our data show that non-specific stress pepsin-related stimuli in primary HHPC are not capable of accelerating Tp63 transcriptional activation, demonstrated by low transcriptional levels of Tp63 in pepsin-treated HHPC (Fig 5B-b), while exhibiting a positive linear relationship to reduced RELA(p65) and significant inverse correlation to Tp53 (Fig 6A, 6B, 6E and 6F). On the other hand, we show that HHK exposed to pepsin produces higher transcriptional levels of Tp63, compared to their corresponding controls but a trend of decreased transcriptional activity at low pH (Fig 5B-a). These data in HHK are supported by the identified inverse correlation between transcriptional levels of Tp63 and RELA(p65) [51], but a significant positive linear correlation to Tp53 with apoptotic function (Fig 6C, 6D, 6E and 6F) [46,52].

The data appear to demonstrate that the combination of pepsin and acid rather than weakly acidic or neutral-pepsin is more toxic *in vitro* and that transcriptional activity may be linked to levels of cell survival. While the observation of decreased cell viability by physiologic pepsin 0.1 mg/ml at pH 4.0 in part supports the observation of reduced transcriptional oncogenic activity at this concentration, it is equally important to note, that neither does less toxic weakly acidic-pepsin at 0.1 mg/ml, pH 5.0, encourage a robust transcriptional oncogenic response. That lower concentrations of pepsin 0.05 and 0.01 mg/ml, presumed less toxic, likewise fail to enable greater transcriptional activity, supports the overall conclusion that combinations of pepsin and acid interact without significant transcriptional oncogenic activity.

## Conclusion

Chronic stimulation of normal human hypopharyngeal cells in a strongly acidic environment with pepsin leads to cell death. In surviving cells, the combination of pepsin and acid does not generate oncogenic NF-*κ*B activity but preserves transcriptional levels of Tp53 in human hypopharyngeal keratinocytes, related to apoptosis [32]. Exposure of normal human hypopharyngeal cells at a less acidic (pH 5.0 and 7.0) environment induces mild activation of NF-*κ*B, which subsequently leads to a mechanism of either cell-cycle arrest or nonspecific stress controlled cell survival but not to oncogenic activity. Our results emphasize the observation that chronic stimulation of normal human hypopharyngeal cells with combinations of pepsin and acid is not capable of activating the NF-*κ*B related oncogenic pathway *in vitro* but rather induces activation of Tp53, and other stress related molecules. We are planning future investigations using our previously established *in vivo* model [11] to clarify dynamic events in tissue response to selected ranges of acidified pepsin.

## Supporting Information

S1 Table Pepsin induced transcriptional levels of NF-κB related oncogenic pathway in human normal hypopharyngeal cells. **A.** Human hypopharyngeal keratinocytes (HHK) exposed to physiologic concentrations of pepsin (0.1 mg/ml), at pH 4.0, 5.0 and 7.0. **B.** Human hypopharyngeal primary cells (HHPC) exposed to different concentrations of pepsin (0.01, 0.05 and 0.1 mg/ml), at pH 4.0, 5.0 and 7.0. **C.** Relative mRNA expression ratios for each target gene in human hypopharyngeal keratinocytes (HHK) exposed to 0.1 mg/ml pepsin at different pH (4.0. 5.0 and 7.0). **D.** Relative mRNA expression ratios for each target gene in human hypopharyngeal primary cells (HHPC) exposed to 0.01, 0.05 and 0.1 mg/ml pepsin, at different pH (4.0. 5.0 and 7.0).
(DOCX)

## Abbreviations

HHPC: Human hypopharyngeal primary cells
HHK: Human hypopharyngeal keratinocytes
