# Supplementary material for: The In Vitro Effect of Acidic-Pepsin on Nuclear Factor KappaB Activation and Its Related Oncogenic Effect on Normal Human Hypopharyngeal Cells
Source: PLoS One. 2016 Dec 14;11(12):e0168269. doi: 10.1371/journal.pone.0168269 (PMC5156414; doi:10.1371/journal.pone.0168269)
Supplement: S1 Table — A. Human hypopharyngeal keratinocytes (HHK) exposed to physiologic concentrations of pepsin (0.1 mg/ml), at pH 4.0, 5.0 and 7.0. B. Human hypopharyngeal primary cells (HHPC) exposed to different concentrations of pepsin (0.01, 0.05 and 0.1 mg/ml), at pH 4.0, 5.0 and 7.0. C. Relative mRNA expression ratios for each target gene in human hypopharyngeal keratinocytes (HHK) exposed to 0.1 mg/ml pepsin at different pH (4.0. 5.0 and 7.0). D. Relative mRNA expression ratios for each target gene in human hypopharyngeal primary cells (HHPC) exposed to 0.01, 0.05 and 0.1 mg/ml pepsin, at different pH (4.0. 5.0 and 7.0). (DOCX) [file pone.0168269.s001.docx]

S1 Table: **Pepsin induced transcriptional levels of NF-κB related oncogenic pathway in human normal hypopharyngeal cells.**

**A.** Human hypopharyngeal keratinocytes (HHK) exposed to physiologic concentrations of pepsin (0.1 mg/ml), at pH 4.0, 5.0 and 7.0.

| **Target gene/**  **hGAPDH**  **(*ΔΔ^CT^*)** | **pH 4.0** | | | | **pH 5.0** | | | | **pH 7.0** | | | |  |
| --- | --- | --- | --- | --- | --- | --- | --- | --- | --- | --- | --- | --- | --- |
|  | **Cntl** | **Pepsin** | | | **Cntl** | **Pepsin** | | | **Cntl** | **Pepsin** | | | **Inactivated Pepsin** |
|  |  | **0.01*** | **0.05** | **0.1** |  | **0.01** | **0.05** | **0.1** |  | **0.01** | **0.05** | **0.1** |  |
| **c-Rel** | 1.22E-01 | 5.13E-01 | 1.26E-02 | 5.26E-03 | 5.86E-02 | 2.09E-01 | 7.71E-02 | 2.09E-02 | 8.71E-02 | 2.44E-01 | 1.06E-01 | 2.44E-02 | 1.70E-02 |
| **RELA(p65)** | 3.00E-03 | 4.05E-04 | 5.00E-04 | 5.03E-05 | 3.07E-04 | 5.81E-04 | 3.90E-04 | 3.56E-04 | 3.83E-04 | 5.69E-04 | 3.55E-04 | 2.07E-04 | 3.00E-04 |
| **bcl-2** | 2.36E-01 | 2.21E-01 | 2.11E-01 | 9.25E-02 | 1.02E-01 | 8.95E-02 | 8.95E-02 | 8.74E-03 | 1.08E-01 | 1.38E-01 | 1.38E-01 | 6.93E-02 | 7.06E-02 |
| **EGFR** | 2.94E-02 | 5.27E-04 | 1.53E-04 | 6.42E-04 | 8.91E-03 | 3.19E-06 | 2.25E-07 | 9.27E-09 | 1.25E-03 | 9.74E-05 | 3.92E-04 | 5.79E-05 | 3.24E-05 |
| **Tp53** | 1.85E-01 | 9.10E-04 | 9.12E-03 | 8.90E-01 | 1.59E-02 | 2.31E-05 | 2.00E-05 | 1.92E-02 | 8.48E-01 | 1.77E-05 | 3.10E-03 | 1.62E-06 | 2.02E-03 |
| **Tp63** | 1.80E-02 | 2.05E-03 | 2.52E-04 | 3.00E-04 | 3.57E-03 | 2.32E-03 | 1.87E-03 | 1.96E-03 | 5.23E-03 | 4.51E-03 | 4.19E-03 | 3.67E-03 | 1.26E-03 |
| **STAT3** | 7.84E-01 | 8.93E-05 | 8.93E-05 | 3.94E-04 | 1.56E-01 | 1.01E-01 | 1.11E-01 | 1.02E-06 | 2.28E-01 | 1.83E-01 | 1.78E-01 | 1.80E-01 | 8.11E-02 |
| **TNFα** | 1.80E+00 | 1.50E-02 | 1.40E-02 | 1.37E-02 | 9.97E-03 | 2.89E-05 | 1.38E-02 | 2.93E-02 | 1.33E-02 | 1.29E-02 | 1.19E-02 | 9.04E-03 | 1.67E-02 |
| **Wnt5α** | 9.26E-02 | 3.44E-03 | 3.44E-05 | 3.22E-04 | 2.84E-03 | 2.12E-05 | 2.07E-05 | 7.22E-08 | 1.24E-01 | 1.10E-04 | 1.81E-03 | 9.24E-03 | 3.09E-02 |

| **Target gene/*h*GAPDH**  **(*ΔΔ^CT^*)** | **pH 4.0** | | **pH 5.0** | | **pH 7.0** | | **Inactivated Pepsin** |
| --- | --- | --- | --- | --- | --- | --- | --- |
|  | **Cntl** | **Pepsin*** | **Cntl** | **Pepsin** | **Cntl** | **Pepsin** |  |
| **c-Rel** | 1.04E-02 | 4.03E-03 | 8.21E-03 | 4.05E-03 | 1.22E-02 | 1.22E-02 | 6.07E-03 |
| **RELA(p65)** | 2.95E-02 | 1.26E-02 | 1.56E-02 | 1.56E-02 | 8.14E-03 | 1.62E-02 | 1.35E-02 |
| **bcl-2** | 4.60E-04 | 7.00E-05 | 7.00E-05 | 7.00E-05 | 1.20E-04 | 1.80E-04 | 1.20E-04 |
| **EGFR** | 1.23E-02 | 3.63E-03 | 5.84E-03 | 8.65E-03 | 7.07E-03 | 9.16E-03 | 5.29E-03 |
| **Tp53** | 1.74E-03 | 7.44E-03 | 6.32E-03 | 7.85E-03 | 1.04E-02 | 9.74E-03 | 5.62E-03 |
| **Tp63** | 2.30E-03 | 1.68E-02 | 2.99E-02 | 3.33E-02 | 4.69E-02 | 6.81E-02 | 3.93E-02 |
| **STAT3** | 1.06E-01 | 9.62E-03 | 2.00E-02 | 1.63E-02 | 1.69E-02 | 2.12E-02 | 1.98E-02 |
| **TNFα** | 2.06E-03 | 1.20E-04 | 6.00E-05 | 3.00E-05 | 5.46E-06 | 2.00E-05 | 2.00E-05 |
| **Wnt5α** | 2.26E-03 | 1.20E-04 | 1.00E-05 | 4.00E-05 | 2.00E-05 | 2.00E-05 | 2.00E-05 |

*0.1 mg/ml

**B.** Human hypopharyngeal primary cells (HHPC) exposed to different concentrations of pepsin (0.01, 0.05 and 0.1 mg/ml), at pH 4.0, 5.0 and 7.0.

*mg/ml

**C.** Relative mRNA expression ratios for each target gene in human hypopharyngeal keratinocytes (HHK)

exposed to 0.1 mg/ml pepsin at different pH (4.0. 5.0 and 7.0).

| **Target gene/**  ***h*GAPDH (*ΔΔ^CT^*)** | **Pepsin/Cntl** | | | **Inactivated pepsin/Cntl** |
| --- | --- | --- | --- | --- |
|  | **pH 4.0** | **pH 5.0** | **pH 7.0** |  |
| **c-Rel** | 3.89E-01 | 4.93E-01 | 1.00E+00 | 4.97E-01 |
| **RELA (p65)** | 4.26E-01 | 1.00E+00 | 1.99E+00 | 1.66E+00 |
| **bcl2** | 1.52E-01 | 1.00E+00 | 1.50E+00 | 1.00E+00 |
| **EGFR** | 2.95E-01 | 1.48E+00 | 1.30E+00 | 7.48E-01 |
| **Tp53** | 4.28E+00 | 1.24E+00 | 9.34E-01 | 5.39E-01 |
| **Tp63** | 7.31E+00 | 1.11E+00 | 1.45E+00 | 8.38E-01 |
| **STAT3** | 9.07E-02 | 8.15E-01 | 1.26E+00 | 1.17E+00 |
| **TNFα** | 5.83E-02 | 5.00E-01 | 3.66E+00 | 3.66E+00 |
| **WNT5α** | 5.31E-02 | 4.00E+00 | 1.00E+00 | 1.00E+00 |

**D.** Relative mRNA expression ratios for each target gene in human hypopharyngeal primary cells (HHPC) exposed to 0.01, 0.05 and 0.1 mg/ml pepsin, at different pH (4.0. 5.0 and 7.0).

| **Target gene/**  ***h*GAPDH (*ΔΔ^CT^*)** | **Pepsin/Cnt (pH 4.0)** | | | **Pepsin/Cnt/ (pH 5.0)** | | | **Pepsin/Cntl (pH 7.0)** | | | **Inactivated pepsin** |
| --- | --- | --- | --- | --- | --- | --- | --- | --- | --- | --- |
|  | **0.1** | **0.05** | **0.1** | **0.01** | **0.05** | **0.1** | **0.01** | **0.05** | **0.1** |  |
| **c-Rel** | 4.20E+00 | 1.03E-01 | 4.31E-02 | 3.56E+00 | 1.31E+00 | 3.56E-01 | 2.81E+00 | 1.22E+00 | 2.81E-01 | 1.95E-01 |
| **RELA (p65)** | 1.35E-01 | 1.67E-01 | 1.68E-02 | 1.89E+00 | 1.27E+00 | 1.16E+00 | 1.49E+00 | 9.27E-01 | 5.40E-01 | 7.83E-01 |
| **bcl2** | 9.35E-01 | 8.92E-01 | 3.92E-01 | 8.76E-01 | 8.76E-01 | 8.55E-02 | 1.28E+00 | 1.28E+00 | 6.42E-01 | 6.53E-01 |
| **EGFR** | 1.79E-02 | 5.20E-03 | 2.18E-02 | 3.58E-04 | 2.52E-05 | 1.04E-06 | 7.79E-02 | 3.14E-01 | 4.63E-02 | 2.59E-02 |
| **Tp53** | 4.92E-03 | 4.93E-02 | 4.81E+00 | 1.46E-03 | 1.26E-03 | 1.21E+00 | 2.09E-05 | 3.65E-03 | 1.91E-06 | 2.38E-03 |
| **Tp63** | 1.14E-01 | 1.40E-02 | 1.67E-02 | 6.49E-01 | 5.24E-01 | 5.48E-01 | 8.63E-01 | 8.01E-01 | 7.01E-01 | 2.40E-01 |
| **STAT3** | 1.14E-04 | 1.14E-04 | 5.03E-04 | 6.49E-01 | 7.13E-01 | 6.52E-06 | 8.01E-01 | 7.82E-01 | 7.90E-01 | 3.56E-01 |
| **TNFα** | 8.33E-03 | 7.78E-03 | 7.61E-03 | 2.90E-03 | 1.38E+00 | 2.94E+00 | 9.65E-01 | 8.90E-01 | 6.78E-01 | 1.26E+00 |
| **WNT5α** | 3.71E-02 | 3.71E-04 | 3.48E-03 | 7.46E-03 | 7.29E-03 | 2.54E-05 | 8.87E-04 | 1.46E-02 | 7.45E-02 | 2.49E-01 |
